# Laparoscopic surgery in abdominal trauma: a single center review of a 7-year experience

**DOI:** 10.1186/s13017-015-0007-8

**Published:** 2015-03-12

**Authors:** Kyoung Hoon Lim, Bong Soo Chung, Jong Yeol Kim, Sung Soo Kim

**Affiliations:** Department of Surgery, Kyungpook National University Hospital, School of Medicine, Kyungpook National University, 50, Samduk-dong 2ga, Jung-gu, Daegu South Korea; Andong General Hospital, Department of Surgery, Andong, South Korea

**Keywords:** Laparoscopy, Therapeutic laparoscopy, Blunt abdominal trauma, Penetrating abdominal trauma

## Abstract

**Introduction:**

Laparoscopic surgery has greatly improved surgical outcome in many areas of abdominal surgery. But many concerns of safety have limited its application in abdominal trauma. We hypothesized that laparoscopy could be safe and efficacious in treatment of patients with abdominal trauma, and reduce the laparotomy related complications (i.e. wound infection, pain, or long hospital stay) as avoiding unnecessary laparotomy.

**Methods:**

From January 2006 to August 2012, a total of 111 patients underwent emergent surgical exploration (laparoscopic, 41; open laparotomy, 70) in Andong General Hospital. Of the 41 patients subjected to laparoscopy, 30 patients had suffered blunt trauma, the remaining 11 patients had sustained penetrating trauma. 31 patients were treated exclusively by laparoscopy and 10 patients underwent laparoscopy-assisted surgery.

**Results:**

The conversion rate was 18%. Major complication was none without postoperative mortality. Comparing laparoscopic surgery with open laparotomy, lesser wound infection, early gas passage, and shorter hospital stay. Otherwise operative times were similar, and neither approach was complicated by missed injury or postoperative intra-abdominal abscess.

**Conclusions:**

Laparoscopic surgery can be performed safely whether injuries are blunt or penetrating, given hemodynamic stability and proper technique. Patients may thus benefit from the shorter hospital stays, greater postoperative comfort (less pain), quicker recoveries, and low morbidity/mortality rates that laparoscopy affords.

## Introduction

Laparoscopy has greatly improved surgical outcomes in many areas of elective abdominal surgery. In acute care surgery, laparoscopy is becoming widely accepted and used with significant advantages in the majority of ACS patients in certain centers with specific experience and laparoscopic skills [1]. However, a number of safety issues have limited its application in abdominal trauma [2]. Due to a high rate of missed injuries, laparoscopy was not well-received for diagnostic evaluation of trauma to the abdomen. However, equipment improvements over time and growing experience on the part of surgeons have overcome former misgivings with respect to penetrating abdominal injuries. Laparoscopy has being slowly attempted as a diagnostic tool for such patients, provided they are hemodynamically stable [3,4]. Under these circumstances, laparoscopic surveillance has been shown to reduce the negative laparotomy rate [5-8]. On the other hand, its utility in patients sustaining blunt abdominal trauma has received only minor attention [9], and the therapeutic role of laparoscopy in trauma patients is still evolving. It was our contention that laparoscopy could be safe and efficacious in both diagnosis and treatment of patients with abdominal trauma, eliminating unnecessary laparotomies and the risks attached.

## Methods

Medical records from the trauma registry at Andong General Hospital were reviewed retrospectively between January, 2006 and August, 2012. A total of 111 patients required surgical exploration for abdominal trauma in this time frame. For patients with penetrating injuries, breach of the peritoneum was grounds for surgical exploration. In patients with blunt injuries, those with unexplained free fluid/air by computed tomography (CT) or worrisome clinical signs and symptoms (ie, evidence of peritoneal irritation, tachycardia, and leukocytosis) were typically evaluated surgically. Five surgeons, each well-trained in colorectal, upper gastrointestinal, or hepatobiliary laparoscopy, performed the collective procedures. Laparoscopy was used at the discretion of the attending trauma surgeon, regardless of the nature of trauma (blunt or penetrating), but hemodynamic stability was mandatory. Therefore, to match two groups, 15 patients that had preoperative hemodynamic instability were excluded in the open group.

Demographic and clinical data retrieved included the type of injury, hemodynamic status on admission, indication for surgery, operative findings, therapeutic procedures performed, need for conversion (to laparotomy), Injury Severity Score (ISS), Sum of abdominal Abbreviated Injury Scale (AIS), presence of peritonitis, operative time, postoperative complications, and mortality. Complications of note were wound infection (requiring delayed closure), anastomotic leak, bleeding with reoperation, missed injury, and postoperative intra-abdominal abscess development. Written informed consent was obtained from the patient for publication of this report and any accompanying images.

### Laparoscopic techniques

**Figure 1:**
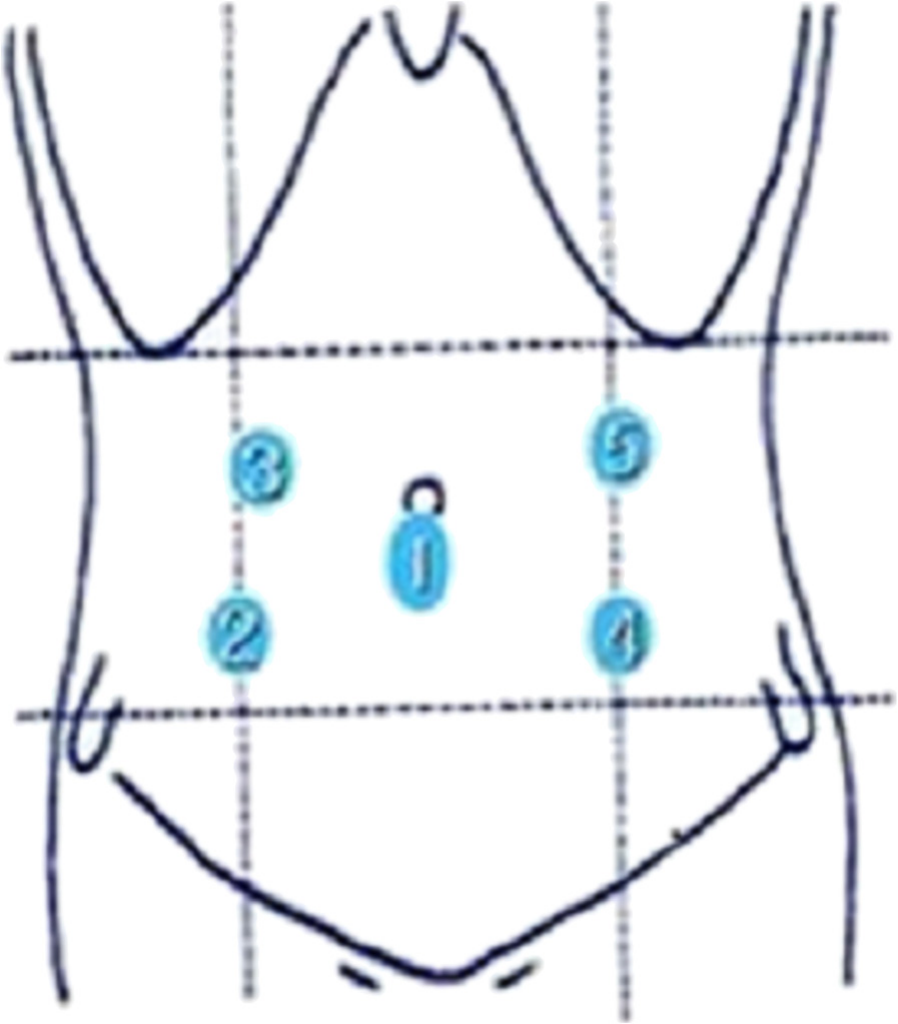
**Laparoscopic trocar entries in abdominal trauma.** ① Umbilical port for laparoscope (10-mm). ② Working port, right iliac fossa (5-mm or 12-mm). ③ Paramedial assist port, right upper quadrant (5-mm). ④ Optional port (5-mm). ⑤ Optional port (5-mm or 12-mm).

**Figure 2:**
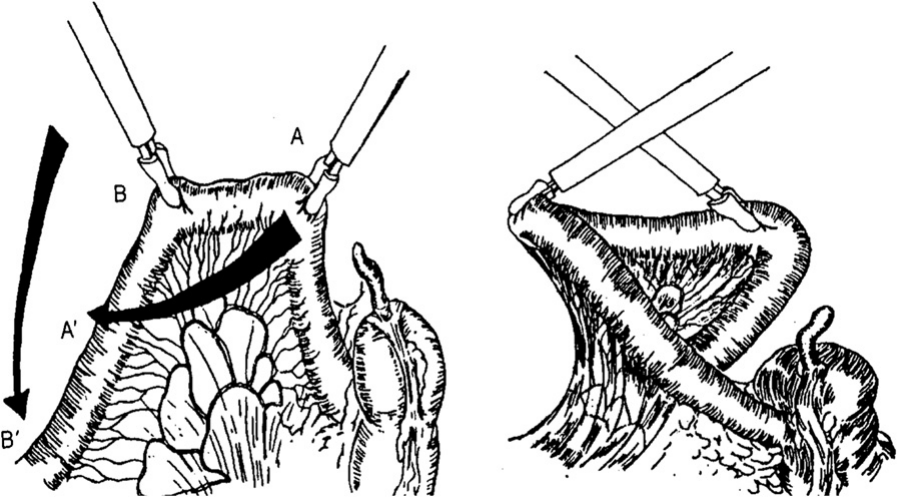
**Elevation of small bowel via atraumatic graspers, with twisting to inspect both aspects of bowel wall and mesentery [10].**

Initially, a 10-mm trocar was inserted via infraumbilical incision. A pneumoperitoneum was then created, using carbon dioxide to induce and maintain (at 12 mmHg) pressure. A 0-degree angle, 10-mm laparoscope was generally used for abdominal exploration. Two additional 5-mm laparoscopic ports were also placed under direct vision at right iliac fossa and at right upper quadrant (paramedial area), with mirror-image ports on the left as needed (Figure 1). Upon insertion of the laparoscope, a search for blood, bile, or intestinal contents was done. Standard examination included inspection of the spleen and liver for bleeding, a check for hollow viscus injury from stomach to rectum, and assessment of small bowel from Treitz’s ligament to ileocecal valve. Using atraumatic bowel graspers, small bowel and mesentery were elevated and appraised in segments. By crossing the graspers, the reverse sides were similarly viewable [10] (Figure 2). This approach was repeated until reaching ileocecal valve, at which point colon was inspected from cecum to rectum. Ultimately, the lesser sac was pierced (through gastrocolic ligament), allowing visualization of posterior gastric wall and most of pancreas (body and tail).

**Figure 3:**
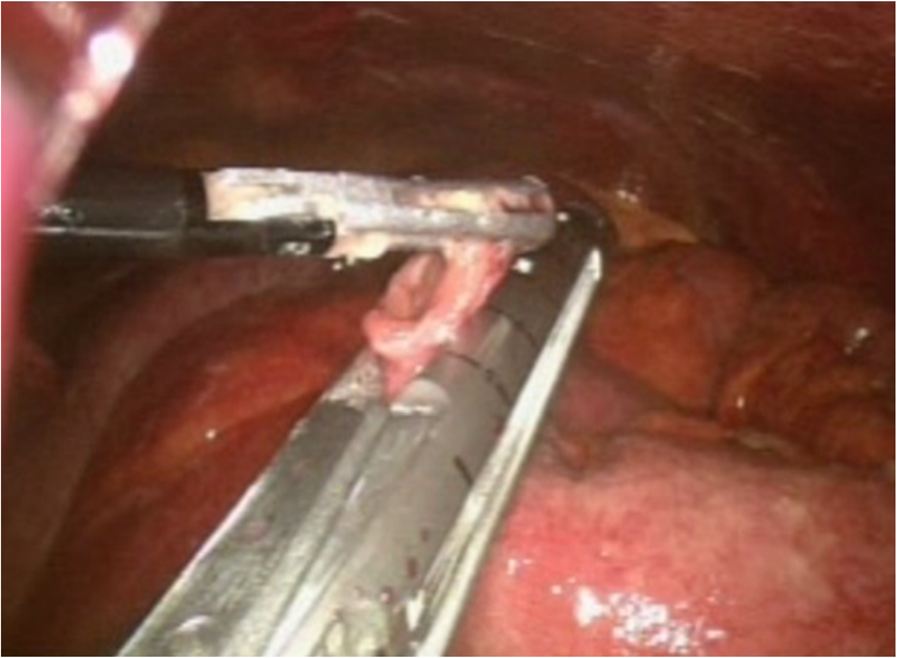
**Stapling of perforated small bowel.**

**Figure 4:**
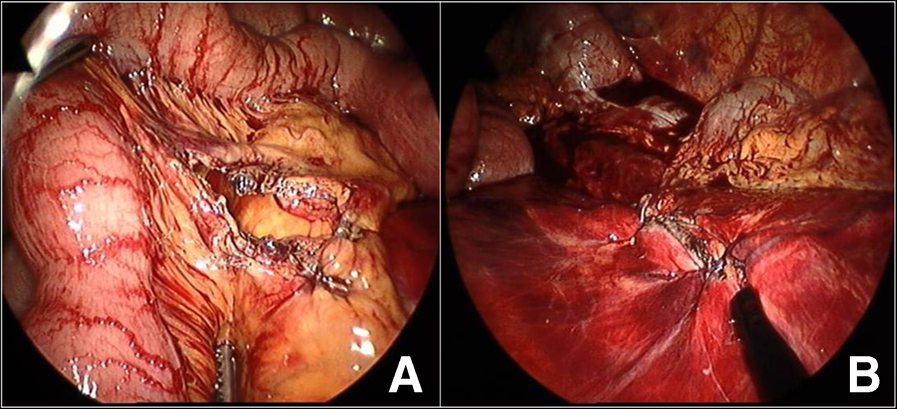
**Control of bleeding from mesenteric tears. A**. Cauterization by Ligasure, **B**. Suturing of torn mesentery.

**Figure 5:**
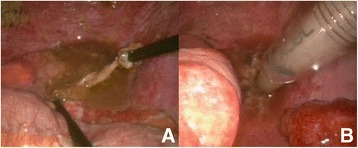
**Methods of evacuation. A**. Large particulate intestinal contents defying conventional endo-sucton, **B**. complete evacuation of large particles via silastic tube, directly inserted through 12-mm port.

Any bowel perforations detected were simply sutured (3–0 vicryl or silk) or closed by linear stapling (endo-GIA®) in the course of the procedure (Figure 3). If segmental resection was needed, a mini-laparotomy was performed by extending the umbilical port to permit laparoscopy-assisted extracorporeal surgery. Bleeding from torn mesentery was controlled by suture ligation or cauterization (Ligasure® or Harmonic scalpel®) (Figure 4). For large volumes of spilled soilage or hematoma (mostly clots) not amenable to aspiration by conventional mode of endo-suction, evacuation was achieved by direct insertion of a silastic tube through a 12-mm port (Figure 5).

### Statistical analysis

Statistical analysis relied on standard windows software (SPSS v20, Chicago, IL), expressing group variables as mean ± standard deviation. Student’s **t**-test was applied to independent samples of continuous variables, whereas chi-square or Fisher’s exact test was used for categorical values. Statistical significance was set at *p* < 0.05.

## Results

**Figure 6:**
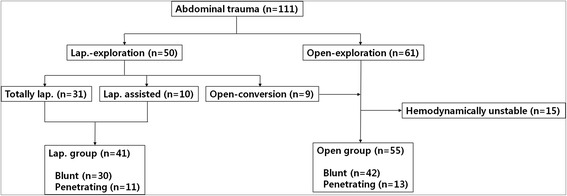
**Flow chart summary of patients.**

In a 7-year period, 111 patients underwent surgery for abdominal trauma. Of these, 41 patients (36.9%) retained the hemodynamic stability required for a laparoscopic procedure and subsequent analysis. Laparoscopy alone was sufficient in 31 (75.6%) instances, whereas 10 patients underwent laparoscopy-assisted procedures. The other 70 patients (63.1%) were treated by open laparotomy, including any conversions (Figure 6). 15 patients of open laparotomy were excluded due to hemodynamic instability for the comparison between laparoscopy and open laparotomy. Therefore, we analyzed laparoscopic group (n = 41) and open group (n = 55).

### Causes of abdominal trauma

Of the 41 patients subjected to laparoscopy, 30 patients (73.2%) had suffered blunt trauma, largely as a consequence of traffic accidents. The remaining 11 patients had sustained penetrating trauma, primarily stab injuries (Table 1).

**Table 1:** **Causes of trauma in patients undergoing surgical intervention**

**Causes of abdominal trauma**	**Laparoscopic**	**Open**
Blunt trauma	30 (73.2)	42 (76.4)
Traffic accident	23 (56.2)	33 (60.0)
Fall	3 (7.3)	5 (9.1)
Work-related injury	3 (7.3)	2 (3.6)
Violence	1 (2.4)	2 (3.6)
Penetrating trauma	11 (26.8)	13 (23.6)
Stab injury	10 (24.4)	13 (23.6)
Gunshot	1 (2.4)	0 (0)

### Injured organs

With blunt trauma, small bowel perforation was most common, followed by torn mesentery. Three patients suffering penetrating trauma also had colon or small bowel perforations. Hemoperitoneum often resulted from injuries of omentum, mesentery, abdominal wall, or spleen (Table 2).

**Table 2:** **Injured organs stratified by injury type**

**Injured organs**	**Laparoscopic**	**Open**
Blunt trauma	30 (73.2)	42 (76.4)
Small bowel	19 (46.3)	27 (49.1)
Mesentery	7 (17.1)	10 (18.2)
Omentum	2 (4.9)	0 (0)
Spleen, liver	2 (4.9)	2 (3.6)
Colon	0 (0)	2 (3.6)
Bladder	0 (0)	1 (1.9)
Penetrating trauma	11 (26.8)	13 (23.6)
Omentum	3 (7.3)	1 (1.8)
Mesentery	2 (4.9)	5 (9.1)
Abdominal wall	2 (4.9)	0 (0)
Colon	2 (4.9)	1 (1.8)
Spleen, liver	1 (2.4)	2 (3.6)
Small bowel	1 (2.4)	4 (7.3)

### Methods of operation

In the 31 patients treated exclusively through laparoscopy, simple closures with suture or stapling (endo-GIA®) sufficed for 13 patients, whereas 11 patients required hemostasis using Ligasure®, Harmonic scalpel®, or suture. If segmental resection was needed for multiple perforations or transection of bowel or for intestinal ischemic changes due to mesenteric tearing, a laparoscopy-assisted mini-laparotomy was performed (Table 3).

**Table 3:** **Operative procedures in patients undergoing surgery**

**Operative procedures**	**Patients (%)**
Exclusively laparoscopic	31 (75.6)
Simple closure (suture, endo-GIA)	13 (31.7)
Bleeding control (suture, Ligasure®)	11 (26.8)
Irrigation & drainage (liver, spleen, pancreas)	4 (9.8)
Examination only (stab injury)	2 (4.9)
Loop colostomy	1 (2.4)
Laparoscopy-assisted (mini-laparotomy)	10 (24.4)
Segmental resection of small bowel	10 (24.4)
Open laparotomy	55 (100)
Simple closure (suture)	25 (45.5)
Bleeding control	18 (32.7)
Segmental resection of small bowel	11 (20.0)
Loop colostomy	1 (1.8)

### Conversion to open laparotomy

The rate of conversion to open laparotomy was 18% (9/50). In early attempts, three laparoscopic procedures were done for diagnosis only. Reasons for conversion were uncontrolled bleeding, voluminous hematoma or spilled bowel contents, massive adhesions from prior surgery, and poor visibility due to edematous bowel (Table 4).

**Table 4:** **Reasons for converting to open laparotomy**

**Reasons for open conversion**	**Patients**
Diagnostic laparoscopy only	3
Uncontrolled bleeding	2
Voluminous hematoma	1
Adhesions from prior surgery	1
Soilage in large amount	1
Edematous bowel (poor visibility)	1

### Comparisons between laparoscopic and open surgery

Comparing laparoscopic surgery with open laparotomy (excluding 15 patients with hemodynamic instability), The parameters presenting severity (ISS, Sum of abdominal AIS, and the presence of peritonitis) were not different between two groups. Wound infection necessitating delayed closure occurred with significantly greater frequency after open laparotomy, and other temporal parameters (time to passage of gas and hospital stay) of open laparotomy were prolonged. Otherwise, respective operative times were similar, and neither approach was complicated by missed injury or postoperative intra-abdominal abscess (Table 5).

**Table 5:** **Open laparotomy and laparoscopic surgery comparison by outcomes**

	**Open (n = 55)**	**Laparoscopic (n = 41)**	***p*** **-value**
Age	57.2 ± 15.6	53.8 ± 15.7	0.296
ISS	9.07 ± 2.8	9.32 ± 3.6	0.708
Sum of abdomen AIS	3.16 ± 0.9	3.17 ± 1.4	0.977
Presence of peritonitis	35 (64%)	23 (56%)	0.529
Operative time (min)	97.2 ± 31.0	91.2 ± 34.6	0.374
Gas passage (day)	2.98 ± 0.9	2.44 ± 0.9	0.006
Hospital stay (day)	17.58 ± 12.7	11.5 ± 5.3	0.004
Complications			
Wound infection	5	0	0.000
Postoperative abscess	0	0	-
Mortality	0	0	-

## Discussion

It is generally upheld that patients who undergo laparoscopy, rather than conventional open surgery, are privy to quicker recovery, less pain, and faster resumption of normal daily routines [11,12]. Laparoscopy is thus a universal choice for elective abdominal surgery. However, a number of concerns have limited its application in abdominal trauma.

Until recently, the presence of peritonitis was a perceived contraindication for laparoscopy, based on the theoretical risk of malignant hypercapnia and toxic shock syndrome. The presumptive risk of malignant hypercapnia implies greater carbon dioxide absorption in the presence of severe intra-abdominal infection and inflammation of the peritoneum; and the danger of toxic shock syndrome is based on potential passage of bacteria and toxins into circulation, due to increased intraperitoneal pressure [13,14]. However, numerous reports of successful outcomes in duodenal and colonic perforation [15-17] soon followed the ground-breaking laparoscopic treatment of a perforated peptic ulcer with peritonitis [18,19]. Unfortunately, treatment of traumatic peritonitis and hemoperitoneum is where laparoscopic surgery has lagged. We have found the above concerns unwarranted with either approach.

Two publications in the 1920s were the first to suggest use of laparoscopy in diagnosing traumatic hemoperitoneum or for detecting blood from traumatic rupture of a viscus [20]. However, the modern concept of diagnostic laparoscopy for trauma was proffered in the 1960s by Heselson [21-23], who reported a series of 68 victims of trauma. In this cohort, laparoscopy was performed to detect hemoperitoneum, penetration of parietal peritoneum, and injury to abdominal organs. Thus the safety, efficacy, and economic benefits of laparoscopy, such as reduced hospitalization time and avoidance of unnecessary laparotomies, were demonstrated. Although infrequently reported, laparoscopy has also served as a therapeutic tool in selected trauma scenarios [24,25], to include the following: autotransfusion of hemoperitoneum [26]; stapling or suturing of small-intestinal wounds; stapling or suturing of stomach and diaphragmatic injuries [24]; splenorrhaphy, hepatorrhaphy, cautery, and topical hemostasis of spleen and liver injuries [24,25,27,28]; laparoscopy-assisted sigmoid colostomy [29]; and application of Ligaclips® to control mesenteric bleeding [30].

When first used for trauma, laparoscopy resulted in high rates of missed injury (41-77%), generating considerable criticism [31]. One of the most serious concerns was its lack of consistency in detecting small bowel damage [4,31,32], which is the main reason surgeons still hesitate today; but because these studies involved both prospective and retrospective analyses and procedures were not standardized, the data are difficult to interpret. In addition, the learning curve of laparoscopic surgery was ignored in early evaluations, and subjective preferences do seem to drive decisions during laparoscopy. One prior report underscored the reliability of laparoscopy as a tool for evaluating traumatic injuries, when used for specific indications and with appropriate technique [24,33,34]. Choi [10] and Kawahara, et al. [8] devised systematic laparoscopic explorations of the abdomen that resulted in no missed injuries. In accordance with the method of Choi, we found it relatively easy to effectively inspect all abdominal organs, without missing an injury.

The primary limitation of laparoscopic intervention is the poor visibility conferred by excessively edematous bowel or uncontrolled active bleeding at presentation. These are the major motivations for conversion to open laparotomy. Edema of the bowel is a time-dependent process. Thus, patients presenting shortly after the traumatic event are more easily managed through laparoscopy, whereas lengthier time intervals usually portend severe intestinal edema. Not only is the laparoscopic window obscured by edematous bowel, but traction injury is more likely to occur during manipulation. One patient in our study was converted to open laparotomy on the basis of intestinal edema. On admission, sedation should be administered for the mechanical ventilation required by respiratory failure due to massive lung contusion, so the initial evaluation of abdomen was omitted, unfortunately. finally, bowel perforation was overlooked initially and was detected three days later after reducing a sedative.

Another cause of open conversion is the spillage of large-sized particulates that cannot be aspirated via the usual mode of endo-suction. However, we were able to achieve complete evacuation in this event by direct insertion of a silastic tube through a 12-mm port. Subsequently, our fears of postoperative intra-abdominal abscess never materialized. A fair number of our open conversions stemmed from trial-and-error in early experience, contributing to an open conversion rate of 18% (9/50). In three patients, the laparoscopies were done for diagnostic purposes only. Two patients with uncontrolled splenic bleeding were converted, as well as two others where large volumes of hematoma and spilled soilage were encountered. With more experience, these conversions very well could have been avoided.

Traumatic abdominal injury is traditionally subject to open exploration and remains a challenge for the general surgeon, especially with respect to controlling wound-related complications. Wound complications still play a major role in lengthy hospital stays and may lead to other delayed morbidities. Our aim was to extend the benefits of minimally invasive surgery to traumatic abdominal injury, thereby decreasing postoperative complications. Indeed, wound infections requiring delayed closure were limited to five patients following open laparotomy. By comparison, none of the patients undergoing laparoscopy suffered a wound complication.

Various temporal parameters (ie, time to passage of gas and hospital stay) were also comparatively better with laparoscopy, although some qualification is needed. Hospital stay was difficult to determine as a function of abdominal surgery in a setting of combined injuries (musculoskeletal, cerebrovascular, pulmonary, etc.). Therefore, we defined hospital stay by points at which oral intake was possible and wound healing was complete.

In conclusion, although we disclose that this study was many limitations caused by selection bias and retrospective study, laparoscopy gradually has being accepted as a diagnostic and/or treatment modality for penetrating abdominal injuries in patients that are hemodynamically stable. The relative rates of morbidity/mortality, postoperative complications, and missed injury are low and compare favorably with an open approach. However, laparoscopic surgery can be performed safely whether injuries are blunt or penetrating, given hemodynamic stability and proper technique. Patients may thus benefit from the shorter hospital stays, greater postoperative comfort (less pain), quicker recoveries, and low morbidity/mortality rates that laparoscopy affords.

## Abbreviations

CT: Computed tomography
ISS: Injury severity score
AIS: Abbreviated injury scale
